# Supplementary material for: COVID-19 Prevalence among Czech Dentists
Source: Int J Environ Res Public Health. 2021 Nov 27;18(23):12488. doi: 10.3390/ijerph182312488 (PMC8656522; doi:10.3390/ijerph182312488)
Supplement: Supplementary file 1 [file ijerph-18-12488-s001.zip › SM.pdf]

COVID-19 prevalence among Czech dentists

Extended version of chapters 3.4.2., 3.4.3., and 3.8.

3.4.2.COVID-19 prevalence based on sex

Sex-based COVID-19 prevalence is provided in Figure 5. Detailed data about the answers provided are available within Supplementary Material.

Out of the 1871 respondents who selected the female sex option, 1246 (66.6%) replied that they were not diagnosed with COVID-19, 467 (24.9%) replied that they were diagnosed with COVID-19, 8 (0.4%) repeatedly, and 130 (6.9%) did not know. A positive test for COVID-19 with no symptoms was admitted by 20 respondents (1.1%). The total female positivity was 26.4%.

Out of the 837 respondents who selected the male sex option, 540 (64.5%) replied that they were not diagnosed with COVID-19, 175 (20.9%) replied that they were diagnosed with COVID-19, 7 (0.8%) repeatedly, and 102 (12.2%) did not know. A positive test for COVID-19 with no symptoms was admitted by 13 respondents (1.6%). The total male positivity was 23.3%. The difference in positivity between the sexes was not statistically significant (Figure 5).

3.4.3.COVID-19 prevalence based on age

Age-based COVID-19 prevalence is illustrated in Figure 6. Age and sex-based COVID-19 prevalence is shown in Figure 7. Detailed data about the answers provided are available within Supplementary Material.

Out of the 187 respondents who selected age up to 30 years of age, 116 (62.0%) replied that they were not diagnosed with COVID-19, 50 (26.7%) replied that they were diagnosed with COVID-19, 0 repeatedly, and 17 (9.1%) did not know. A positive test for COVID-19 with no symptoms was admitted by 4 respondents (2.1%). %). The total positivity in this group was 28.9%.

Out of the 713 respondents who selected age between 30-40 years, 431 (60.4%) replied that they were not diagnosed with COVID-19, 193 (27.1%) replied that they were diagnosed with COVID-19, 7 (1.0%) repeatedly, and 73 (10.2%) did not know. A positive test for COVID-19 with no symptoms was admitted by 9 respondents (1.3%). The total positivity in this group was 29.3%.

Out of the 446 respondents who selected age between 40-50 years, 266 (59.6%) replied that they were not diagnosed with COVID-19, 123 (27.6%) replied that they were diagnosed with COVID-19, 1 (0.2%) repeatedly, and 47 (10.5%) did not know. A positive test for COVID-19 with no symptoms was admitted by 9 respondents (2.0%). %). The total positivity in this group was 29.8%.

Out of the 479 respondents who selected age between 50-60 years, 324 (67.6%) replied that they were not diagnosed with COVID-19, 114 (23.8%) replied that they were diagnosed with COVID-19, 2 (0.4%) repeatedly, and 36 (7.5%) did not know. A positive test for COVID-19 with no symptoms was admitted by 3 respondents (0.6%). %). The total positivity in this group was 24.8%.

Out of the 740 respondents who selected age between 60-70 years, 536 (72.4%) replied that they were not diagnosed with COVID-19, 143

(19.3%) replied that they were diagnosed with COVID-19, 4 (0.5%) repeatedly, and 51 (6.9%) did not know. A positive test for COVID-19 with no symptoms was admitted by 6 respondents (0.8%). %). The total positivity in this group was 20.7%.

Out of the 187 respondents who selected age over 70 years, 116 (62.0%) replied that they were not diagnosed with COVID-19, 50 (26.7%) replied that they were diagnosed with COVID-19, 0 repeatedly, and 17 (9.1%) did not know. A positive test for COVID-19 with no symptoms was admitted by 4 respondents (2.1%). %). The total positivity in this group was 15.2% (Figure 6 and 7).

3.8. Environment where the infection occurred

This question was addressed only to the respondents who reported that they knew or suspected where they were infected within Q8 (n = 518). Out of them, 517 (99.8%) respondents answered this question

A total of 243 (47.0%) respondents replied they were infected in their household environment, 92 (17.8%) by a patient at their work, 53 (10.2%) by another team member at work, 54 (10.4%) at work without knowing from whom, 36 (7.0%) were infected in another environment despite following all epidemiological regulations, 22 (4.3%) were infected in another environment admitting that they had not complied with all epidemiological regulations, and 17 (3.3%) chose an open answer that could not be classified into any of the previous categories.

These results show that 199 (38.4%) respondents identified the work environment as a source of infection. Together with the domestic environment (47.0%), these two categories were the dominant source of infection among respondents, jointly responsible for 85.5% of the reported transmission (Figure 12).
